# Investigation of Hydro-Lipophilic Properties of *N*-Alkoxyphenylhydroxynaphthalenecarboxamides †

**DOI:** 10.3390/molecules23071635

**Published:** 2018-07-04

**Authors:** Iva Kapustikova, Andrzej Bak, Tomas Gonec, Jiri Kos, Violetta Kozik, Josef Jampilek

**Affiliations:** 1 Department of Pharmaceutical Chemistry, Faculty of Pharmacy, Comenius University in Bratislava, Odbojarov 10, 83232 Bratislava, Slovakia; jirikos85@gmail.com (J.K.); josef.jampilek@gmail.com (J.J.); 2 Department of Synthesis Chemistry, Faculty of Mathematics, Physics and Chemistry, University of Silesia, Szkolna 9, 40007 Katowice, Poland; violetta.kozik@us.edu.pl; 3 Department of Chemical Drugs, Faculty of Pharmacy, University of Veterinary and Pharmaceutical Sciences Brno, Palackeho 1, 61242 Brno, Czech Republic; t.gonec@seznam.cz

**Keywords:** hydroxynaphthalenecarboxamides, lipophilicity determinations, structure-lipophilicity relationships

## Abstract

The evaluation of the lipophilic characteristics of biologically active agents is indispensable for the rational design of ADMET-tailored structure–activity models. *N*-Alkoxy-3-hydroxynaphthalene-2-carboxanilides, *N*-alkoxy-1-hydroxynaphthalene-2-carboxanilides, and *N*-alkoxy-2-hydroxynaphthalene-1-carboxanilides were recently reported as a series of compounds with antimycobacterial, antibacterial, and herbicidal activity. As it was found that the lipophilicity of these biologically active agents determines their activity, the hydro-lipophilic properties of all three series were investigated in this study. All 57 anilides were analyzed using the reversed-phase high-performance liquid chromatography method for the measurement of lipophilicity. The procedure was performed under isocratic conditions with methanol as an organic modifier in the mobile phase using an end-capped non-polar C_18_ stationary reversed-phase column. In the present study, a range of software lipophilicity predictors for the estimation of clogP values of a set of *N*-alkoxyphenylhydroxynaphthalenecarboxamides was employed and subsequently cross-compared with experimental parameters. Thus, the empirical values of lipophilicity (log*k*) and the distributive parameters (π) were compared with the corresponding in silico characteristics that were calculated using alternative methods for deducing the lipophilic features. To scrutinize (dis)similarities between the derivatives, a PCA procedure was applied to visualize the major differences in the performance of molecules with respect to their lipophilic profile, molecular weight, and violations of Lipinski’s Rule of Five.

## 1. Introduction

The assessment of a molecular property profile that is crucial for the bioavailability of compounds and hence critical for the prospective drug candidate is possible by an a priori calculation of molecular descriptors. The ADMET-tailored properties are essentially estimated based on the molecular structure as ‘intuitive roadmaps’ even before the synthesis of the molecule has been rationalized. Molecular descriptors quantifying drug-like properties are easily calculated based on the molecular formula; however, the reliability of the resulting values of such properties is still questionable. The empirical analysis of the marketed drug-size library provided a heuristic guide (Lipinski’s Rule of Five (Ro5) ‘sieve’) that relates the calculable physicochemical properties to the complex in vivo pharmacokinetic parameter that comprises aqueous solubility and oral bioavailability. In fact, the Ro5 restricts the ‘drug-like property space’ through a set of threshold values, but a good drug-like score does not necessarily make a drug [1,2,3].

Lipophilicity is known as a first-rate physicochemical parameter that is increasingly important in the description of both pharmacokinetic (ADMET) and pharmacodynamic aspects of drug–receptor/enzyme interactions, which often correlate well with the bioactivity of chemicals [4,5,6]. Understanding lipophilicity and its modulation has been recognized as a crucial factor for the successful passage of a drug through clinical development; therefore, its quantitative descriptor (logP) is considerably used at the early stages of drug development [7,8,9,10]. This thermodynamic parameter (logP) describes the partitioning of a compound between an aqueous and an organic (octanol) phases, and can be characterized by the partition coefficient [9,10,11,12,13]. The logP is determined for the uncharged species of the drug. Note that it may exist preferably in the ionic or zwitterionic form(s). With new computerized methods of logP calculation, the possibility of predicting the lipophilicity of large libraries of compounds appeared. However, algorithms that are sensitive to various electronic effects and individual structural aspects are still needed.

The existing empirical lipophilicity database is negligible compared to the enormous number of compounds for which such data are desirable. In fact, partitions coefficients have been determined empirically for approximately 30,000 substances (10^4^), which is only a tiny fraction of the factual chemical space (FCS) populations (10^8^)—in this sense, logP is a molecular property that is specified in pretty rare cases. The accurate and efficient measurement of lipophilicity is an important requirement in drug design, as the created database can be used for logP estimation for millions of hypothetical molecules under design.

Experimental methods for lipophilicity determination can be divided according to their principle into two groups: shake-flask partitioning and chromatographic methods (reversed-phase thin layer chromatography, reversed-phase high-performance liquid chromatography (RP-HPLC), and centrifugal partition chromatography). Additionally, alternative experimental methods can be mentioned, such as, e.g., slow stirring, flow-injection extraction, or pH-metric logP determination. Shake-flask partitioning is the most classical method. The principle of this method is the partitioning of a compound between one of a wide variety of lipid phases and an aqueous phase (water or buffer solution), while concentrations of the compound in the individual phases are most frequently determined by ultraviolet-visible spectrophotometry, HPLC, gas chromatography, or potentiometric (pH-meter probe). Although historically shake-flask partitioning is considered “the gold standard”, there are several related problems, such as the high amount of sample required (low concentration of sample alludes to a detection limit), microemulsion forming (which prevents the separation of the two layers), instability (decomposition), or impurities precluding the determination of real concentrations and the requirement to validate this complicated system. Thus, it can be stated that the experimental procedures for the estimation of the partition coefficient are basically time-consuming and/or material-consuming, and require a high purity of the solute [8,9,10].

It was recognized long ago that the retention of a compound in reversed-phase (RP) liquid chromatography is governed by its lipophilicity, and thus shows correlation with the octanol–water partition coefficient [14]. In RP chromatography, hydrophobic forces govern the retention, and it was recognized as a potential method for lipophilicity determination [15,16,17,18,19]. HPLC provides an excellent platform for computer-controlled automated measurements with computerized data acquisition for a large number of research compounds. Other advantages in the use of the HPLC retention data for lipophilicity determination are as follows: there is no need for the determination of concentration or method validation; small impurities are separated from the main component; small amounts of the material are needed for measurements; and the measurements can be completely automated. Therefore, the investigation of the true potential of this method is of great importance [20]. Reviews on the effects of stationary and mobile phase selection were published, e.g., by van der Waterbeemd [9] or by Claessens [21]. RP-HPLC methods have become popular and widely used for lipophilicity measurement [22]. The general procedure involves measuring a directly accessible retention time under isocratic conditions with varying amounts of an organic modifier in the mobile phase using end-capped non-polar C_18_ stationary RP columns, and calculating the capacity factor *k* [8,9,23,24,25,26]. Log*k* is the logarithm of the capacity factors in chromatographic approaches, which is related to the partitioning of a compound between a mobile and a (pseudo)stationary phase. Log*k* is then used as the lipophilicity index converted to the logP scale [9,20,21,22,27,28].

In addition to this method, alternative lipophilicity descriptors have been proposed using mainly in silico predictive models, e.g., the Hansch π constant derived for chemical constituents as an additive property, where logP is calculated by summing contributions from structural fragments [7]. It is possible that some methods for the theoretical calculation of lipophilicity might be more or less suitable for a specific/heterogeneous series of analyzed compounds; thus, a variety of approaches should be employed in a particular *consensus* methodology, and subsequently compared with the existing empirical data [29].

*N*-Alkoxy-3-hydroxynaphthalene-2-carboxanilides, *N*-alkoxy-1-hydroxynaphthalene-2-carbox-anilides, and *N*-alkoxy-2-hydroxynaphthalene-1-carboxanilides were recently synthesized and tested for their antibacterial and antimycobacterial activity, as well as for their activity related to the inhibition of photosynthetic electron transport (PET) in spinach (*Spinacia oleracea* L.) chloroplasts [30,31,32,33,34,35]. Since it was found that the lipophilicity of these significantly biologically effective agents determined their activity, the hydro-lipophilic properties of all three series are investigated in this study. The primary objective of the current study was to investigate a range of various software logP predictors for the estimation of numerical lipophilic values of the ensemble of *N*-alkoxyphenylhydroxynaphthalenecarboxamide derivatives with a subsequent cross-comparison with experimental parameters. Thus, the empirical lipophilicity (log*k*) was compared with the corresponding logP characteristics that were calculated using alternative methods for deducing the lipophilic features. The mean values of the selected molecular descriptors that average over the chosen calculation methods were subsequently correlated with the log*k* parameter in *consensus* clogP.

## 2. Results and Discussion

### 2.1. Chemistry

**Scheme 1:**
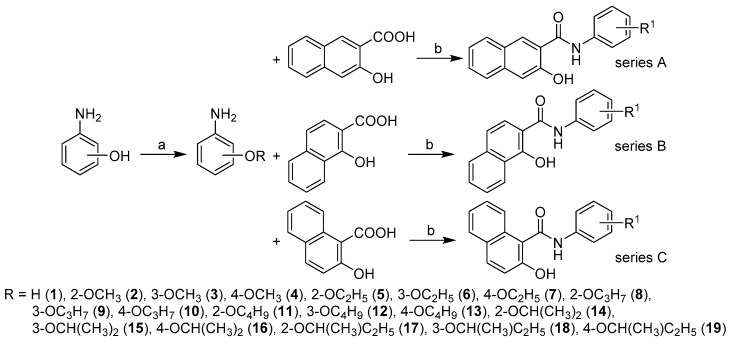
Synthesis of *N*-substituted 3-hydroxynaphthalene-2-carboxanilides **1a**–**19a** (series A), *N*-substituted 1-hydroxynaphthalene-2-carboxanilides **1b**–**19b** (series B), and *N*-substituted 2-hydroxynaphthalene-1-carboxanilides **1c**–**19c** (series C). Reagents and conditions: (**a**) R-Br, NaH, acetonitrile, room temperature, 24 h [31]; (**b**) PCl_3_, chlorobenzene, MW, 15 min [30,31,32,33,34].

The condensation of hydroxynaphthalenecarboxylic acids with the appropriate alkoxy-substituted anilines using phosphorus trichloride in dry chlorobenzene under microwave conditions gave series A of *N*-substituted 3-hydroxynaphthalene-2-carboxanilides **1a**–**19a** in yields ranging from 52% to 80% [30,31], series B of *N*-substituted 1-hydroxynaphthalene-2-carboxanilides **1b**–**19b** in yields ranging from 49% to 86% [32,34], and series C of *N*-substituted 2-hydroxynaphthalene-1-carboxanilides **1c**–**19c** in yields ranging from 43% to 87% [33,34]. Unique commercially unavailable alkoxy-substituted anilines (i.e., except *o-*anisidine, *m-*anisidine, and *p-*anisidine) were prepared by a modified procedure according to De Marco et al. [36] using the direct alkylation of corresponding aminophenols by alkyl bromides in the presence of sodium hydride as reported recently [31], see Scheme 1. The yields of individual alkoxy-substituted anilines were as follows: 2-/3-/4-ethoxyanilines 70–80%, 2-/3-/4-propoxyaniline 63–73%, 2-/3-/4-butoxyaniline 68–79%, 2-/3-/4-(prop-2-yloxy)aniline 52–62%, and 2-/3-/4-(but-2-yloxy)aniline 56–64% [31].

### 2.2. Consensus Lipophilicity Estimation

**Figure 1:**
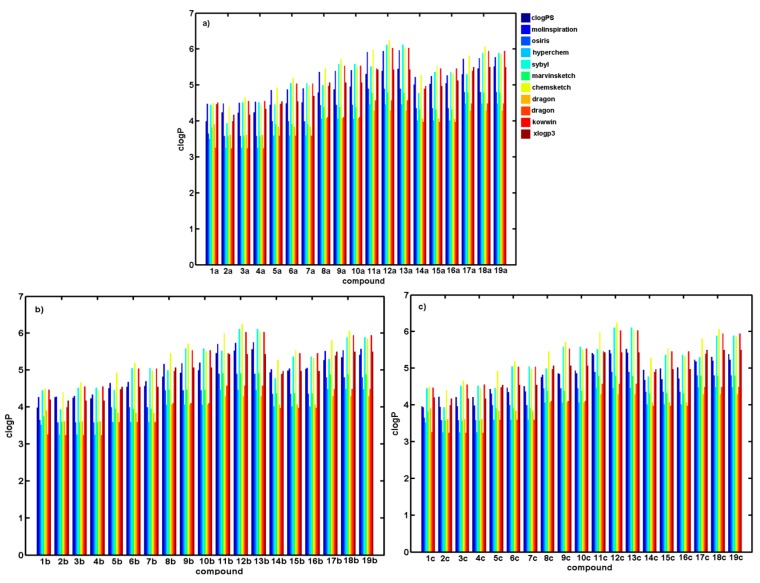
Theoretical partition coefficient calculated by alternative methods for (**a**) *N*-alkoxyphenyl-3-hydroxynaphthalene-2-carboxanilides **1a**–**19a** (series A), (**b**) *N*-alkoxyphenyl-1-hydroxynaphthalene-2-carboxanilides **1b**–**19b** (series B), and (**c**) *N*-alkoxyphenyl-2-hydroxynaphthalene-1-carboxanilides **1c**–**19c** (series C).

A range of various software logP predictors (clogPS, Molinspirations, OSIRIS, HyperChem 7.0, Sybyl X, MarvinSketch 15, ACD/ChemSketch 2015, Dragon6.0, Kowwin, and XlogP3) for the estimation of numerical lipophilic values was employed for a set of analyzed derivatives divided into three series (positional isomers A, B, and C) and subsequently cross-compared with the experimental parameters. Thus, the empirical capacity factor (*k*) was compared with the corresponding logP characteristics that were calculated using alternative methods for deducing lipophilic features. The numerical values of the theoretical partition coefficients for positional isomers specified by distinct in silico principles are listed in Table 1, Table 2 and Table 3, and illustrated in Figure 1. In fact, most of the programs, except for clogPS, Molinspiration, and MarvinSketch software, do not distinguish between the calculated lipophilicity values of positional isomers. Not surprisingly, within particular groups, lipophilicity increases with the number of carbon atoms in the side chain, as observed for unbranched (**1**–**13**) and branched (**14**–**19**) subseries, respectively. Moreover, some variations in logP values are probably related to a different algorithm (atom/fragment/descriptor-based) that was implemented in the software predictors and training data used (see Section 3.3 and Scheme S1 in the Supplementary materials).

**Figure 2:**
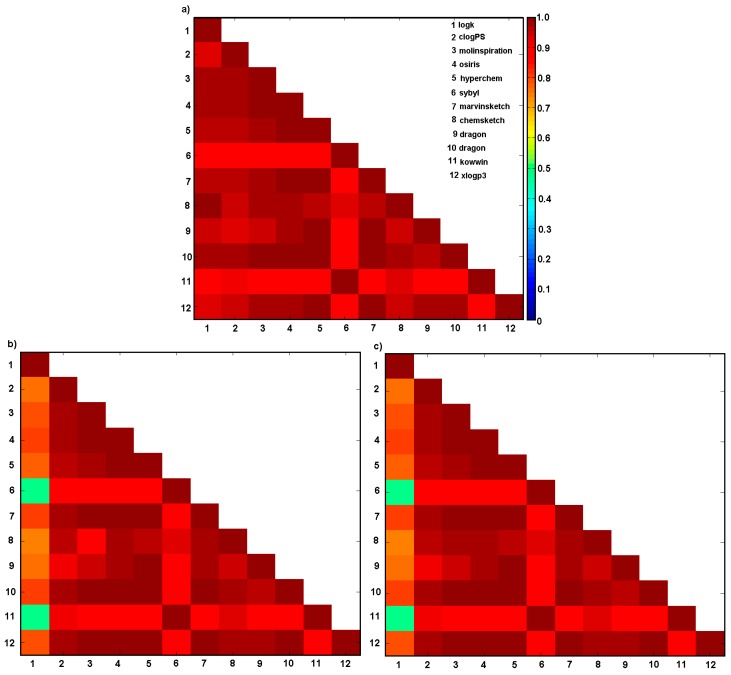
Matrix of correlation coefficients of linear relationships between experimental lipophilicity log*k* (1) and calculated lipophilicity with clogPS (2), Molinspirations (3), OSIRIS property explorer (4), HyperChem 7.0 (5), Sybyl X (6), Marvin Sketch (ChemAxon) 15 (7), ChemSketch 2015 (8), Dragon 6.0 (9), Dragon 6.0 (10), Kowwin (11), XlogP3 (12) methods for series (**a**) **1a**–**19a**, (**b**) **1b**–**19b** and (**c**) **1c**–**19c**.

A relatively high cross-correlation was revealed within the predicted values of logP for the analyzed set of compounds, as shown by the triangular matrix of linear correlation parameters in Figure 2 and Tables S1–S3 (Supplementary materials). On the other hand, a lower (cross-)correlation was revealed within the predicted values of logP for all 57 anilides (series A, B, and C) as listed in Table S4, and illustrated by the triangular matrix of linear correlation parameters in Figure S1 (Supplementary materials).

Moreover, the collected data indicate a relevant correlation between the experimental lipophilicity (log*k*) and calculated logP values for series A with r ranging from 0.87 to 0.99. A worse match was depicted for series B and C, as illustrated in Figure 2b,c, respectively. In fact, the poor predictive performance of clogP specified by Sybyl and Kowwin software can be partially explained by the insufficient coverage of the chemical space by the measured compounds; models are as good as the data they are based on. Additionally, it seems that intramolecular interactions play a vital role in lipophilicity estimation where the closeness of the second benzene nucleus of the naphthalene scaffold to the phenolic moiety (series B) or the amide moiety (series C) cannot be neglected.

The backward elimination with the IVE-PLS procedure employed for the clogP matrix (X_19×11_) and the log*k* parameter as a dependent variable (Y_19×1_) for each series of compounds indicated that MarvinSketch, ChemSketch, XlogP3, Sybyl, and Kowwin property predictors contribute significantly to the final model. It is noteworthy that the balanced selection of clogP estimators prevents the overfitting phenomenon by covering the vast spectrum of theoretical procedures, rather than only the best (inter)correlated. The mean values of the selected molecular descriptors that average over the chosen calculation methods were subsequently correlated with the log*k* parameters, namely *consensus* clogP, with correlation coefficients of 0.95 for series A, 0.73 for series B, and 0.64 for series C.

### 2.3. Descriptor-Based Similarity Assessment

In an additional experiment, the PCA procedure for an ensemble of descriptors retrieved from DRAGON 6.0 software was applied to the analyzed compounds. From the initial number of selected parameters (4885), all of the columns with constant or nearly constant values (standard deviation < 10^−4^) and with missing values have been excluded at the preprocessing stage, resulting in the final set of 2645 descriptors. The final dataset was arranged in a matrix X_57×2645_ with rows representing molecules called objects, and columns presenting numerical parameters called variables. Principal component analysis (PCA) was employed to visualize relevant variations within the entire set of molecules with respect to their structure and lipophilicity profile. The analysis was performed for centered and standardized values, since the studied data library includes parameters of various orders of magnitude. The percentage of the modeled variance was a relevant factor in the determination of the model complexity (number of principal components (PCs)).

**Figure 3:**
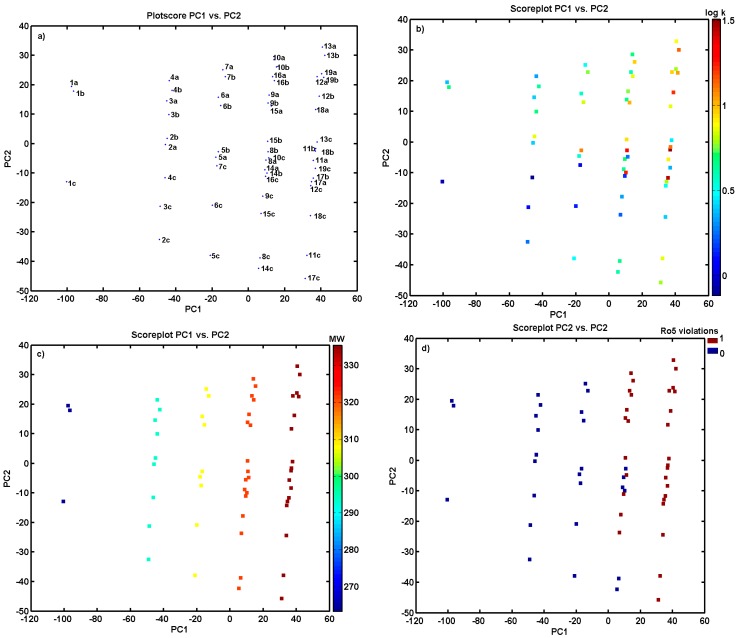
Projection of derivatives **1a**–**19c** on plane defined by first versus second principal component (**a**) with: log*k* (**b**), molecule´s weight, (**c**) and number of Lipinski’s Ro5 violations (**d**). Colors code the values of experimental lipophilicity, molecular weight, and the number of Ro5 violations.

The PCA model with the first four PCs described 80.78% of the total data variance, while the first two PCs accounted for 67.90%. The analysis of the score plots PC1 versus PC2 in Figure 3a indicates that the investigated analogues can be classified into groups according to structural parameters; the positional isomers are generally grouped together. PC1, which describes 52.07% of the total variance, reveals the major variations between compounds **1a**–**c** that are unmodified structures (only hydrogen as an R substituent within a phenyl ring), and the remaining ones. Interestingly, positional isomers of group C are noticeably separated from the A and B series along the PC2 < −10, indicating (dis)similarities between the studied objects. The inspection of the experimental lipophilicity, color-coded accordingly to experimental log*k* values for objects projected on the plane specified by two first principal components (PC1 versus PC2), confirmed that group C is basically prescribed with lower empirical lipophilicity, as illustrated in Figure 3b. Obviously, the consequence of higher lipophilicity is the vulnerability of increased molecular weight (MW), which is termed ‘molecular obesity’. In fact, positional isomers are not differentiated according to MW values, as plotted in Figure 3c. Accordingly, the molecule lipophilic profile is in line with the number of Lipinski’s Ro5 violations, as depicted in Figure 3d for compounds **9**–**19** in series A, B, and C.

### 2.4. Experimental Lipophilicity and Biological Effects

**Figure 4:**
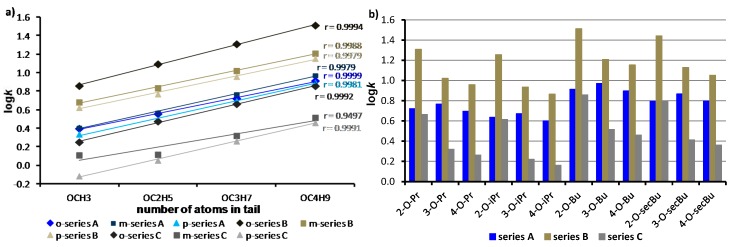
Comparison of experimentally determined the log*k* values of three discussed series of *N*-alkoxy substituted compounds: 3-hydroxynaphthalene-2-carboxanilides **2a**–**19a** (series A), 1-hydroxynaphthalene-2-carboxanilides **2b**–**19b** (series B), and 2-hydroxynaphthalene-1-carboxanilides **2c**–**19c** (series C): only unbranched alkoxy chains (**a**) and branched alkoxy chains and their unbranched isomers (**b**).

Based on experimentally determined log*k* values, it can be stated that lipophilicity logically linearly (with correlation factors ranging from 0.9497 to 0.9999; *n* = 4) increases with the lengthening of the unbranched (OCH_3_ < OC_2_H_5_ < OC_3_H_7_ < OC_4_H_9_) alkoxy tail (see Figure 4a). Nevertheless, it should be noted that unsubstituted compounds **1a**–**c** showed higher experimental lipophilicity than compounds **4a**–**c** (R = 4-OCH_3_), as shown in Table 1. Branched alkoxy substituents, i.e., compounds **14a**–**c**, **15a**–**c**, **16a**–**c** (R = OCH(CH_3_)_2_), and **17a**–**c**, **18a**–**c**, and **19a**–**c** (R = OCH(CH_3_)CH_2_CH_3_) showed lower lipophilicity than their unbranched *n*-alkoxy isomers **8a**–**c**, **9a**–**c**, **10a**–**c,** and **11a**–**c**, **12a**–**c**, **13a**–**c** (see Figure 4b), which corresponds to our previously reported results [37,38].

Distributive parameters π have been firmly established as parameters of choice for correlating binding to biological macromolecules and transport through a biological system. The constant π describes the lipophilicity contribution of individual moieties substituted into some skeleton [39,40]. These π parameters characterizing the hydrophobicity of individual substituents were calculated according to the formula π = log*k*_S_ − log*k*_U_, where log*k*_S_ is the determined capacity factor logarithm of individual substituted compounds, while log*k*_U_ is the determined capacity factor logarithm of unsubstituted compounds (i.e., **1a**–**c**); it means π = 0. The π values of individual substituents in the discussed compounds are shown in Table 4.

**Figure 5:**
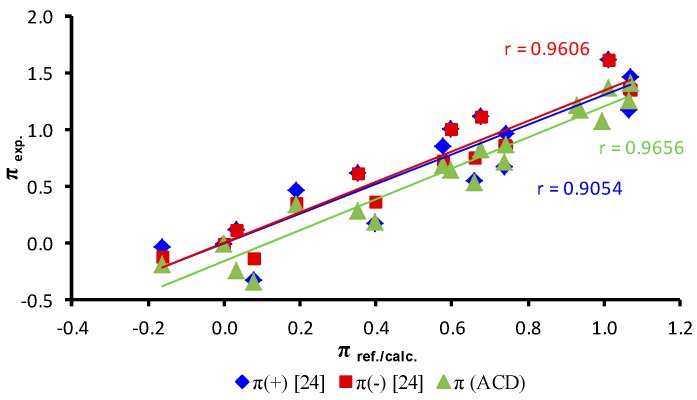
Comparison of values of experimentally determined parameters π of individual alkoxy substituents in *ortho*-, *meta*- and *para*-positions of an anilide ring with parameters π^(+)^, π^(−)^ (ref. [24]) and calculated parameters π (ACD/Percepta).

Distributive constants π of individual substituents are dependent on the scaffold (aliphatic, aromatic, heteroaromatic). A number of distributive parameters of π for various substituents for all three substituent positions in the benzene or heteroaromatic ring were described [41]. In addition, the parameter π is also dependent on other substituents, and parameter π^(−)^ should be used in case of compounds having other electron-donating substituents, while π^(+)^ should be used in case of compounds having other strong electron-withdrawing substituents [39]. The determined π parameters of substituents can be used for describing relationships between physicochemical properties and the biological activity of the prepared *N*-alkoxy-substituted hydroxynaphthalenecarboxanilides. Thus, these determined parameters of π of specific *N*-alkoxy-substituted hydroxynaphthalenecarboxanilides were compared with π^(+)^ and π^(−)^ substituent constants obtained from the literature [39] and calculated by ACD/Percepta ver. 2012 for verification. All of the distributive parameters π^(+)^ and π^(−)^ are shown in Table 4, and the match is illustrated in Figure 5.

Based on the size of the standard deviation values of the individual mean values of parameters π for alkoxy substituents within all three series, it can be stated that the π parameters have predictive merit. From dependences in Figure 5, where experimentally determined parameters π are plotted with parameters π found in literature or calculated by ACD/Percepta, it is evident that experimental parameters π have a good match with π^(−)^ parameters published by Norrington et al. [39] (*r* = 0.9606, *n* = 16) and calculated by ACD/Percepta (*r* = 0.9656, *n* = 19). All of the differences between the experimentally determined π parameters of alkoxy tails and the π parameters calculated or obtained from the literature may be caused, as mentioned above, by interactions of alkoxy chains with amide and, in case of *ortho*- and *meta*-substituents, with the spatially close phenolic moiety.

It can be assumed that the experimentally determined log*k* values or π parameters specify lipophilicity within the individual series of compounds, and can be used as a useful tool for the further investigation of structure–activity relationships within these series of biologically active compounds.

All the above-discussed observations correspond to biological activities; e.g., the lipophilic *N*-(alkoxyphenyl)-1-hydroxynaphthalene-2-carboxamides of series B demonstrated higher potency against non-tuberculous mycobacteria *Mycobacterium smegmatis* and *M. kansasii* than compounds of series A and C, but also a stronger antiproliferative effect against the human monocytic leukemia THP-1 cell line [31,34]. In addition, the compounds of series B significantly affected photosystem II, which resulted in the inhibition of photosynthetic electron transport in spinach (*Spinacia oleracea* L.) chloroplasts [35].

## 3. Experimental Section

### 3.1. Synthesis

The discussed *N*-alkoxyphenylhydroxynaphthalenecarboxamides **1a**–**7c** were synthesized using microwave-assisted synthesis, as described recently by Kos et al. [30] and Gonec et al. [31,32,33,34]. The studied compounds are presented in Table 1.

### 3.2. Lipophilicity Determination by HPLC (Capacity Factor k/Calculated Logk)

A HPLC separation module Waters Alliance 2695 XE equipped with a Waters Dual Absorbance Detector 2486 (Waters Corp., Milford, MA, USA) was used. A chromatographic column Symmetry^®^ C_18_ 5 µm, 4.6 × 250 mm, Part No. W21751W016 (Waters Corp.) was used. The HPLC separation process was monitored by Empower^®^ 3 Chromatography Manager Software (Waters Corp.). Isocratic elution by a mixture of MeOH p.a. (72%) and H_2_O-HPLC Mili-Q grade (28%) as a mobile phase was used. The total flow of the column was 1.0 mL/min, the injection was 20 μL, the column temperature was 40 °C, and the sample temperature was 10 °C. A detection wavelength of 210 nm was chosen. The KI methanolic solution was used for the determination of dead time (t_D_). Retention times (t_R_) were measured in minutes. Capacity factors *k* were calculated using the Empower^®^ 3 Chromatography Manager Software according to the formula *k* = (t_R_ − t_D_)/t_D_, where t_R_ is the retention time of the solute, while t_D_ is the dead time obtained using an unretained analyte. Each experiment was repeated three times. Log*k*, which was calculated from the capacity factor *k*, is used as the lipophilicity index converted to the logP scale. The log*k* values of individual compounds are shown in Table 1, Table 2 and Table 3.

### 3.3. Theoretical Lipophilicity Estimation

The theoretical partition coefficient can be calculated using a vast range of computer programs that are freely or commercially available, for instance (see the values in Table 1, Table 2 and Table 3):

milogP—method developed by Molinspiration, which is able to process practically all of the organic molecules as a sum of the fragment-based contributions and correction factors;

AlogPS—method provided by Tetko et al. [42], which is based on atom-type electrotopological-state (E-state) indices and neural network technology;

ClogP—fragmental procedure implemented in a Sybyl/Centara package provides highly accurate lipophilicity predictions based on structure-dependent correction values taken from Hansch and Leo’s database;

HyperChem logP—an atom-additive method that estimates lipophilicity based on the individual atomic contribution according to Ghose, Prichett, and Crippen;

MarvinSketch logP—the overall lipophilicity of a molecule is composed of the contributing values of its atoms, the types of which were redefined to accommodate electron delocalization and the contributions of ionic forms;

ChemSketch logP—a high quality, versatile fragment-based algorithm with an accuracy provided by models calculated on the basis of experimental data. Well-characterized logP contributions have been compiled for atoms, structural fragments, and intramolecular interactions derived from >12,000 experimental logP values;

Dragon AlogP—the statistical parameters of the Ghose–Crippen–Viswanadhan model were estimated with known experimental logP on a training set of 8364 compounds. The overall value of the lipophilic atomic-based constant is calculated with the contribution of 115 atom types;

Dragon MlogP—the calculated partition coefficient incorporates VdW volume and Moriguchi polar parameters as correction factors. The MlogP model consists of a regression equation based on 13 structural parameters that were evaluated by a training set of 1230 organic molecules;

Kowwin—estimates the log octanol–water partition coefficient of chemicals using an atom/fragment contribution method;

XlogP3—an atom-additive method with well-defined correction factors that implements an optimized atom typing scheme calibrated on a large training set.

Basic methods for in silico lipophilicity specification are illustrated in Scheme S1 (Supplementary materials).

### 3.4. Iterative PLS-Based Variable Elimination

Molecular data encoded by various descriptors usually shows a significant degree of overlap; therefore, the relatively high data uncertainty constitutes an issue in the attempt to assign or predict a particular property. Redundant variables may negatively affect regressive analysis; therefore, a reduction in the number of uninformative variables might unarguably improve PLS models. Although variable elimination in PLS modeling is a complex issue, several procedures have been introduced recently, including iterative variable elimination (IVE-PLS), which was successfully employed in the multidimensional (mD-QSAR) methods [43]. The iterative IVE-PLS procedure is used in the current calculations as an enhancement of the single-step UVE algorithm that was originally proposed by Centner et al. for identification of the variables to be eliminated [44]. Briefly, the whole algorithm is composed of the following steps:

*Step* *1.* Standard PLS analysis with LOO-CV to assess the performance of the PLS model
*Step* *2.* Elimination of a matrix column with the lowest abs(mean(b)/std(b)) value
*Step* *3.* Standard PLS analysis of the new matrix without the column rejected in step 2
*Step* *4.* Recurrent repetition of steps 1–3 to maximize the LOO parameter

### 3.5. Principal Component Analysis

A linear projection procedure called principal component analysis (PCA) condenses a larger multidimensional dataset into a few explanatory linear combinations of the original data called principal components or PC scores (PCs). A restricted set of orthogonal PCs being a linear combination of weighted input vectors forms a basis for the lower-dimensional space, while maintaining the input space topology [45]. The diversity exploration is enabled with calculated distance metrics using the projection of multidimensional objects into the two/three-dimensional PCs space. The PCA model decomposes information contained in a data matrix into scores and loadings, where the score matrix contains information about any similarities among the data objects, while the loading matrix enables similarities among the variables. The PCA model with *f* principal components for a data matrix *X* can be presented as follows: *X = TP^T^ + E,* where *X* is a data matrix with *m* objects and *n* variables, *T* is the score matrix with dimensions (*m × f*), *P^T^* is a transposed matrix of loadings with dimensions (*f × n*), and *E* is a matrix of the residual variance (*m × n*) that is not explained by the first *f* principal components. The PCA usually reduces the number of variables while retaining most of the original information.

## 4. Conclusions

The quantitative evaluation of the lipophilic characteristics of potential drug molecules is indispensable for the rational design of ADMET-tailored structure–activity models; hence, robust algorithms for deriving logP from the molecular structure are needed. Consequently, a range of tools for rapid in silico logP estimation emerged; however, the quality of logP prediction is still questionable. The poor predictive performance of software packages for theoretical lipophilicity determination can be partially explained by insufficient coverage of the chemical space by measured compounds; models are as good as the data they are based on. Compared with the great number of compounds for which such data are desirable, the current experimental data are notably insufficient. It is known that some methods for theoretical lipophilicity specification are more or less suited for specific/heterogeneous series of compounds; therefore, a variety of approaches should be employed and subsequently compared with empirical data. In the current study, a range of software lipophilicity predictors for estimation of clogP values for a set of *N*-alkoxyphenylhydroxynaphthalene-carboxamide derivatives was employed and subsequently cross-compared with the experimental parameters. Thus, the empirical lipophilicity (log*k*) was compared with the corresponding logP characteristics that were calculated using alternative methods for deducing lipophilic features, and relatively high cross-correlation was revealed within the predicted values of logP. Moreover, the collected data indicate a relevant correlation between the experimental lipophilicity (log*k*) and calculated logP values. The mean values of the selected molecular descriptors that average over the chosen calculation methods were subsequently correlated with a log*k* parameter, namely *consensus* clogP. To scrutinize the (dis)similarities between derivatives, a PCA procedure was applied to visualize the major differences in the performance of molecules with respect to their lipophilic profile, molecular weight, and Ro5 violations.

## Figures and Tables

**Table 1:**
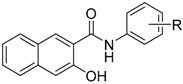
*N*-substituted 3-hydroxynaphthalene-2-carboxanilides **1a**–**19a** (series A), as well as their determined log*k* and theoretical partition coefficients calculated by alternative methods.

No.	log*k*	logP ^a^	miLogP ^b^	ClogP ^c^	ClogP ^d^	ClogP ^e^	ClogP ^f^	ClogP ^g^	MlogP ^h^	AlogP ^i^	ClogP ^j^	ClogP ^k^
**1a**	0.3927	3.99	4.48	3.65	3.51	4.45	3.82	4.50	3.91	3.26	4.47	4.52
**2a**	0.3982	4.24	4.49	3.58	3.25	3.93	3.57	4.40	3.61	3.24	3.99	4.17
**3a**	0.4055	4.23	4.51	3.58	3.25	4.52	3.57	4.66	3.61	3.24	4.55	4.17
**4a**	0.3374	4.24	4.53	3.58	3.25	4.52	3.57	4.45	3.61	3.24	4.55	4.33
**5a**	0.5570	4.45	4.86	3.99	3.59	4.46	3.91	4.93	3.84	3.59	4.48	4.54
**6a**	0.5682	4.49	4.88	3.99	3.59	5.05	3.91	5.19	3.84	3.59	5.04	4.54
**7a**	0.4916	4.52	4.91	3.99	3.59	5.05	3.91	4.98	3.84	3.59	5.04	4.70
**8a**	0.7221	4.79	5.36	4.44	4.06	4.99	4.38	5.46	4.07	4.11	4.97	5.07
**9a**	0.7672	4.88	5.39	4.45	4.06	5.58	4.38	5.72	4.07	4.11	5.54	5.07
**10a**	0.6963	4.95	5.41	4.45	4.06	5.58	4.38	5.51	4.07	4.11	5.54	5.07
**11a**	0.9136	5.31	5.92	4.90	4.46	5.52	4.78	5.99	4.29	4.57	5.46	5.43
**12a**	0.9711	5.39	5.95	4.90	4.46	6.12	4.78	6.25	4.29	4.57	6.03	5.43
**13a**	0.8961	5.45	5.97	4.90	4.46	6.12	4.78	6.04	4.29	4.57	6.03	5.43
**14a**	0.6360	5.01	5.22	4.35	4.01	4.77	4.32	5.28	4.07	3.97	4.90	4.97
**15a**	0.6723	5.03	5.25	4.35	4.01	5.36	4.32	5.54	4.07	3.97	5.46	4.97
**16**	0.6017	5.05	5.27	4.35	4.01	5.36	4.32	5.33	4.07	3.97	5.46	5.13
**17a**	0.7956	5.30	5.73	4.80	4.48	5.30	4.79	5.81	4.29	4.49	5.39	5.50
**18a**	0.8670	5.46	5.75	4.80	4.48	5.89	4.79	6.07	4.29	4.49	5.95	5.50
**19a**	0.7977	5.52	5.77	4.80	4.48	5.89	4.79	5.86	4.29	4.49	5.95	5.50

^a^ clogPS, ^b^ Molinspirations, ^c^ OSIRIS property explorer, ^d^ HyperChem 7.0, ^e^ Sybyl X, ^f^ Marvin Sketch (ChemAxon) 15, ^g^ ChemSketch 2015, ^h^ Dragon 6.0, ^i^ Dragon 6.0, ^j^ Kowwin, ^k^ XlogP3).

**Table 2:**
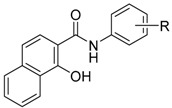
*N*-substituted 1-hydroxynaphthalene-2-carboxanilides **1b**–**19b** (series B), as well as their determined log*k* and theoretical partition coefficients calculated by alternative methods.

No.	log*k*	logP ^a^	miLogP ^b^	ClogP ^c^	ClogP ^d^	ClogP ^e^	ClogP ^f^	ClogP ^g^	MlogP ^h^	AlogP ^i^	ClogP ^j^	ClogP ^k^
**1b**	0.6755	3.98	4.27	3.65	3.51	4.45	3.75	4.50	3.91	3.26	4.47	4.20
**2b**	0.8593	4.27	4.28	3.58	3.25	3.93	3.59	4.40	3.61	3.24	3.99	4.17
**3b**	0.6828	4.25	4.30	3.58	3.25	4.52	3.59	4.66	3.61	3.24	4.55	4.17
**4b**	0.6239	4.23	4.33	3.58	3.25	4.52	3.59	4.45	3.61	3.24	4.55	4.17
**5b**	1.0940	4.51	4.65	3.99	3.59	4.46	3.95	4.93	3.84	3.59	4.48	4.54
**6b**	0.8353	4.54	4.68	3.99	3.59	5.05	3.95	5.19	3.84	3.59	5.04	4.54
**7b**	0.7700	4.56	4.70	3.99	3.59	5.05	3.95	4.98	3.84	3.59	5.04	4.54
**8b**	1.3103	4.82	5.16	4.45	4.06	4.99	4.47	5.46	4.07	4.11	4.97	5.07
**9b**	1.0215	4.93	5.18	4.45	4.06	5.58	4.47	5.72	4.07	4.11	5.54	5.07
**10b**	0.9588	4.99	5.20	4.45	4.06	5.58	4.47	5.51	4.07	4.11	5.54	5.07
**11b**	1.5122	5.46	5.71	4.90	4.46	5.52	4.92	5.99	4.29	4.57	5.46	5.43
**12b**	1.2088	5.53	5.74	4.90	4.46	6.11	4.92	6.25	4.29	4.57	6.03	5.43
**13b**	1.1537	5.56	5.76	4.90	4.46	6.11	4.92	6.04	4.29	4.57	6.03	5.43
**14b**	1.2556	4.94	5.02	4.35	4.01	4.77	4.37	5.28	4.07	3.97	4.90	4.97
**15b**	0.9355	4.98	5.04	4.35	4.01	5.36	4.37	5.54	4.07	3.97	5.46	4.97
**16b**	0.8648	5.03	5.06	4.35	4.01	5.36	4.37	5.33	4.07	3.97	5.46	4.97
**17b**	1.4424	5.27	5.52	4.80	4.48	5.30	4.89	5.81	4.29	4.49	5.39	5.50
**18b**	1.1291	5.35	5.54	4.80	4.48	5.89	4.89	6.07	4.29	4.49	5.95	5.50
**19b**	1.0518	5.41	5.57	4.80	4.48	5.89	4.89	5.86	4.29	4.49	5.95	5.50

^a^ clogPS, ^b^ Molinspirations, ^c^ OSIRIS property explorer, ^d^ HyperChem 7.0, ^e^ Sybyl X, ^f^ Marvin Sketch (ChemAxon) 15, ^g^ ChemSketch 2015, ^h^ Dragon 6.0, ^i^ Dragon 6.0, ^j^ Kowwin, ^k^ XlogP3).

**Table 3:**
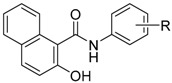
*N*-substituted 2-hydroxynaphthalene-1-carboxanilides **1c**–**19c** (series C), as well as their determined log*k* and theoretical partition coefficients calculated by alternative methods.

No.	log*k*	logP ^a^	miLogP ^b^	ClogP ^c^	ClogP ^d^	ClogP ^e^	ClogP ^f^	ClogP ^g^	MlogP ^h^	AlogP ^i^	ClogP ^j^	ClogP ^k^
**1c**	−0.0581	3.96	3.93	3.65	3.51	4.45	3.82	4.50	3.91	3.26	4.47	4.20
**2c**	0.2518	4.22	3.94	3.58	3.25	3.93	3.57	4.40	3.61	3.24	3.99	4.17
**3c**	0.1106	4.21	3.96	3.58	3.25	4.52	3.57	4.66	3.61	3.24	4.55	4.17
**4c**	−0.1149	4.21	3.98	3.58	3.25	4.52	3.57	4.45	3.61	3.24	4.55	4.17
**5c**	0.4759	4.43	4.31	3.99	3.59	4.46	3.91	4.93	3.84	3.59	4.48	4.54
**6c**	0.1175	4.47	4.34	3.99	3.59	5.05	3.91	5.19	3.84	3.59	5.04	4.54
**7c**	0.0542	4.51	4.36	3.99	3.59	5.05	3.91	4.98	3.84	3.59	5.04	4.54
**8c**	0.6639	4.75	4.82	4.45	4.06	4.99	4.38	5.46	4.07	4.11	4.97	5.07
**9c**	0.3209	4.86	4.84	4.45	4.06	5.58	4.38	5.72	4.07	4.11	5.54	5.07
**10c**	0.2622	4.94	4.86	4.45	4.06	5.58	4.38	5.51	4.07	4.11	5.54	5.07
**11c**	0.8578	5.41	5.37	4.90	4.46	5.52	4.78	5.99	4.29	4.57	5.46	5.43
**12c**	0.5161	5.50	5.40	4.90	4.46	6.11	4.78	6.25	4.29	4.57	6.03	5.43
**13c**	0.4604	5.53	5.42	4.90	4.46	6.11	4.78	6.04	4.29	4.57	6.03	5.43
**14c**	0.6145	4.95	4.68	4.35	4.01	4.77	4.32	5.28	4.07	3.97	4.90	4.97
**15c**	0.2214	4.99	4.70	4.35	4.01	5.36	4.32	5.54	4.07	3.97	5.46	4.97
**16c**	0.1619	5.02	4.72	4.35	4.01	5.36	4.32	5.33	4.07	3.97	5.46	4.97
**17c**	0.7927	5.23	5.18	4.80	4.48	5.30	4.79	5.81	4.29	4.49	5.39	5.50
**18c**	0.4129	5.31	5.20	4.80	4.48	5.89	4.79	6.07	4.29	4.49	5.95	5.50
**19c**	0.3621	5.38	5.23	4.80	4.48	5.89	4.79	5.86	4.29	4.49	5.95	5.50

^a^ clogPS, ^b^ Molinspirations, ^c^ OSIRIS property explorer, ^d^ HyperChem 7.0, ^e^ Sybyl X, ^f^ Marvin Sketch (ChemAxon) 15, ^g^ ChemSketch 2015, ^h^ Dragon 6.0, ^i^ Dragon 6.0, ^j^ Kowwin, ^k^ XlogP3).

**Table 4:** Comparison of determined distributive parameters π calculated from log*k* for individual alkoxy substituents within an individual series of hydroxynaphthalenecarboxanilides A–C, values of distributive parameters π expressed as means ± SD and parameters π of discussed alkoxy substituents obtained from literature [24] and calculated by ACD/Percepta ver. 2012. (N.F. = not found in literature).

Comp.	R	π_A_	π_B_	π_C_	π ± SD	π^+^ [39]	π^−^ [39]	π ^‡^
**1**	H	0	0	0	0	0	0	0
**2**	2-OCH_3_	0.09	0.09	0.05	0.08 ± 0.02	−0.33	−0.13	−0.34
**3**	3-OCH_3_	0.04	0.04	0.02	0.03 ± 0.01	0.12	0.12	−0.24
**4**	4-OCH_3_	−0.15	−0.15	−0.19	−0.16 ± 0.02	−0.03	−0.12	−0.19
**5**	2-OC_2_H_5_	0.40	0.40	0.39	0.40 ± 0.01	0.17	0.37	0.19
**6**	3-OC_2_H_5_	0.36	0.36	0.34	0.35 ± 0.01	0.62	0.62	0.29
**7**	4-OC_2_H_5_	0.15	0.15	0.27	0.19 ± 0.07	0.47	0.35	0.34
**8**	2-OC_3_H_7_	0.74	0.74	0.73	0.74 ± 0.01	0.67	0.87	0.72
**9**	3-OC_3_H_7_	0.69	0.69	0.65	0.68 ± 0.02	1.12	1.12	0.82
**10**	4-OC_3_H_7_	0.75	0.75	0.72	0.74 ± 0.02	0.97	0.85	0.87
**11**	2-OC_4_H_9_	1.08	1.08	1.04	1.07 ± 0.02	1.17	1.37	1.26
**12**	3-OC_4_H_9_	1.02	1.02	1.00	1.01 ± 0.01	1.62	1.62	1.36
**13**	4-OC_4_H_9_	1.08	1.08	1.05	1.07 ± 0.02	1.47	1.35	1.41
**14**	2-OCH(CH_3_)_2_	0.66	0.66	0.66	0.66 ± 0.01	0.55	0.75	0.54
**15**	3-OCH(CH_3_)_2_	0.61	0.61	0.57	0.60 ± 0.02	1.00	1.00	0.64
**16**	4-OCH(CH_3_)_2_	0.59	0.59	0.55	0.58 ± 0.02	0.85	0.73	0.69
**17**	2-OCH(CH_3_)CH_2_CH_3_	1.00	1.00	0.98	0.99 ± 0.01	N.F.	N.F.	1.07
**18**	3-OCH(CH_3_)CH_2_CH_3_	0.95	0.95	0.91	0.94 ± 0.02	N.F.	N.F.	1.17
**19**	4-OCH(CH_3_)CH_2_CH_3_	0.09	0.94	0.91	0.93 ± 0.02	N.F.	N.F.	1.22

**^‡^** Calculated using ACD/Percepta ver. 2012 (Advanced Chemistry Development, Toronto, ON, Canada).

## Supplementary Materials

The supplementary material is available online. Table S1. Matrix of correlation coefficients (n = 19, α = 0.05) of linear relationships between particular partition coefficients and experimental lipophilicity data (logk) for N-substituted 3-hydroxynaphthalene-2-carboxanilides 1a–19a (series A); Table S2. Matrix of correlation coefficients (n = 19, α = 0.05) of linear relationships between particular partition coefficients and experimental lipophilicity data for N-substituted 1-hydroxynaphthalene-2-carboxanilides 1b–19b (series B); Table S3. Matrix of correlation coefficients (n = 19, α = 0.05) of linear relationships between particular partition coefficients and experimental lipophilicity data for N-substituted 2-hydroxynaphthalene-1-carboxanilides 1c–19c (series C); Table S4. Matrix of correlation coefficients (n = 57, α = 0.05) of linear relationships between particular partition coefficients and experimental lipophilicity data for entire ensemble of compounds (series A, B, C); Figure S1. Matrix of correlation coefficients of linear relationships between experimental lipophilicity (log*k*^1^) and calculated lipophilicity with clogPS^2^, Molinspirations^3^, OSIRIS property explorer4, HyperChem 7.0^5^, Sybyl X^6^, Marvin Sketch (ChemAxon) 15^7^, ChemSketch 2015^8^, Dragon 6.0^9^, Dragon 6.0^10^, Kowwin^11^, XlogP3^12^ methods for the entire ensemble of compounds (series A, B, C); Scheme S1. Basic methods for in silico lipophilicity specification.
