# Supplementary material for: Investigation of Hydro-Lipophilic Properties of N-Alkoxyphenylhydroxynaphthalenecarboxamides
Source: Molecules. 2018 Jul 4;23(7):1635. doi: 10.3390/molecules23071635 (PMC6099728; doi:10.3390/molecules23071635)

Supplementary Materials

Investigation of Physicochemical Properties of *N*-Alkoxyphenylhydroxynaphthalene-carboxamides †

Iva Kapustikova [1,*], Andrzej Bak [2,*], Tomas Gonec [3], Jiri Kos [1], Violetta Kozik [2] and Josef Jampilek [1]

[1] Department of Pharmaceutical Chemistry, Faculty of Pharmacy, Comenius University, Odbojarov 10, 83232 Bratislava, Slovakia; jirikos85@gmail.com (J.K.); josef.jampilek@gmail.com (J.J.)

[2] Department of Synthesis Chemistry, Faculty of Mathematics, Physics and Chemistry, University of Silesia, Szkolna 9, 40007 Katowice, Poland; violetta.kozik@us.edu.pl

[3] Department of Chemical Drugs, Faculty of Pharmacy, University of Veterinary and Pharmaceutical Sciences, Palackeho 1, 61242 Brno, Czech Republic; t.gonec@seznam.cz

* Correspondence: kapustikova@fpharm.uniba.sk (I.K.); andrzej.bak@us.edu.pl (A.B.)

† Preliminary results were presented at The 21th Electronic Conference on Synthetic Organic Chemistry (ECSOC-21, http://sciforum.net/conference/ecsoc-21/paper/4719), 1–30 November 2017 (paper 4719).

Table S1. Matrix of correlation coefficients (n=19, α=0.05) of linear relationships between particular partition coefficients and experimental lipophilicity data (log*k*) for *N*-substituted 3-hydroxynaphthalene-2-carboxanilides **1a–19a** (series A).

	**log*k***	**logP[a]**	**miLogP[b]**	**ClogP[c]**	**ClogP[d]**	**ClogP[e]**	**ClogP[f]**	**ClogP[g]**	**MlogP[h]**	**AlogP[i]**	**ClogP[j]**	**ClogP[k]**
**log*k***	1											
**logP[a]**	0.93	1										
**miLogP[b]**	0.98	0.97	1									
**ClogP[c]**	0.98	0.97	0.99	1								
**ClogP[d]**	0.96	0.96	0.98	0.99	1							
**ClogP[e]**	0.88	0.88	0.90	0.89	0.88	1						
**ClogP[f]**	0.96	0.96	0.98	0.99	1.00	0.88	1					
**ClogP[g]**	0.99	0.95	0.98	0.98	0.96	0.93	0.96	1				
**MlogP[h]**	0.94	0.93	0.95	0.97	0.99	0.86	0.99	0.94	1			
**AlogP[i]**	0.98	0.97	0.99	0.99	0.99	0.90	0.99	0.98	0.96	1		
**ClogP[j]**	0.87	0.91	0.90	0.90	0.89	0.99	0.89	0.93	0.87	0.89	1	
**ClogP[k]**	0.93	0.95	0.97	0.98	0.99	0.88	0.99	0.95	0.98	0.98	0.90	1

[a] clogPS, [b] Molinspirations, [c] OSIRIS property explorer, [d] HyperChem 7.0, [e] Sybyl X, [f] Marvin Sketch (ChemAxon) 15, [g] ChemSketch 2015, [h] Dragon 6.0, [i] Dragon 6.0, [j] Kowwin, [k] XlogP3.

Table S2. Matrix of correlation coefficients (n=19, α=0.05) of linear relationships between particular partition coefficients and experimental lipophilicity data for *N*-substituted 1-hydroxynaphthalene-2-carboxanilides **1b–19b** (series B).

	**log*k***	**logP[a]**	**miLogP[b]**	**ClogP[c]**	**ClogP[d]**	**ClogP[e]**	**ClogP[f]**	**ClogP[g]**	**MlogP[h]**	**AlogP[i]**	**ClogP[j]**	**ClogP[k]**
**log*k***	1											
**logP[a]**	0.75	1										
**miLogP[b]**	0.79	0.98	1									
**ClogP[c]**	0.80	0.98	0.99	1								
**ClogP[d]**	0.78	0.96	0.98	0.99	1							
**ClogP[e]**	0.47	0.90	0.90	0.89	0.88	1						
**ClogP[f]**	0.80	0.97	0.99	0.99	0.99	0.88	1					
**ClogP[g]**	0.74	0.96	0.90	0.98	0.96	0.93	0.97	1				
**MlogP[h]**	0.76	0.92	0.95	0.97	0.99	0.86	0.98	0.94	1			
**AlogP[i]**	0.80	0.98	0.99	0.99	0.99	0.89	0.99	0.98	0.96	1		
**ClogP[j]**	0.47	0.91	0.90	0.90	0.89	0.99	0.89	0.93	0.87	0.89	1	
**ClogP[k]**	0.79	0.97	0.99	0.99	0.99	0.88	0.99	0.97	0.97	0.99	0.89	1

[a] clogPS, [b] Molinspirations, [c] OSIRIS property explorer, [d] HyperChem 7.0, [e] Sybyl X, [f] Marvin Sketch (ChemAxon) 15, [g] ChemSketch 2015, [h] Dragon 6.0, [i] Dragon 6.0, [j] Kowwin, [k] XlogP3.

Table S3. Matrix of correlation coefficients (n=19, α=0.05) of linear relationships between particular partition coefficients and experimental lipophilicity data for *N*-substituted 2-hydroxynaphthalene-1-carboxanilides **1c–19c** (series C).

	**log*k***	**logP[a]**	**miLogP[b]**	**ClogP[c]**	**ClogP[d]**	**ClogP[e]**	**ClogP[f]**	**ClogP[g]**	**MlogP[h]**	**AlogP[i]**	**ClogP[j]**	**ClogP[k]**
**log*k***	1											
**logP[a]**	0.75	1										
**miLogP[b]**	0.79	0.98	1									
**ClogP[c]**	0.80	0.98	0.99	1								
**ClogP[d]**	0.78	0.96	0.98	0.99	1							
**ClogP[e]**	0.47	0.90	0.90	0.89	0.88	1						
**ClogP[f]**	0.80	0.97	0.99	0.99	0.99	0.88	1					
**ClogP[g]**	0.74	0.96	0.98	0.98	0.96	0.93	0.97	1				
**MlogP[h]**	0.76	0.92	0.95	0.97	0.99	0.86	0.98	0.94	1			
**AlogP[i]**	0.80	0.98	0.99	0.99	0.99	0.89	0.99	0.98	0.96	1		
**ClogP[j]**	0.47	0.91	0.90	0.90	0.89	0.99	0.89	0.93	0.87	0.89	1	
**ClogP[k]**	0.79	0.97	0.99	0.99	0.99	0.88	0.99	0.97	0.97	0.99	0.89	1

[a] clogPS, [b] Molinspirations, [c] OSIRIS property explorer, [d] HyperChem 7.0, [e] Sybyl X, [f] Marvin Sketch (ChemAxon) 15, [g] ChemSketch 2015, [h] Dragon 6.0, [i] Dragon 6.0, [j] Kowwin, [k] XlogP3.

Table S4. Matrix of correlation coefficients (n=57, α=0.05) of linear relationships between particular partition coefficients and experimental lipophilicity data for entire ensemble of compounds (series A, B, C).

	**log*k***	**logP[a]**	**miLogP[b]**	**ClogP[c]**	**ClogP[d]**	**ClogP[e]**	**ClogP[f]**	**ClogP[g]**	**MlogP[h]**	**AlogP[i]**	**ClogP[j]**	**ClogP[k]**
**log*k***	1											
**logP[a]**	0.52	1										
**miLogP[b]**	0.66	0.90	1									
**ClogP[c]**	0.53	0.97	0.91	1								
**ClogP[d]**	0.52	0.96	0.90	0.99	1							
**ClogP[e]**	0.35	0.89	0.83	0.89	0.88	1						
**ClogP[f]**	0.56	0.96	0.90	0.99	0.99	0.88	1					
**ClogP[g]**	0.50	0.96	0.89	0.98	0.96	0.93	0.96	1				
**MlogP[h]**	0.50	0.92	0.87	0.97	0.99	0.86	0.98	0.94	1			
**AlogP[i]**	0.53	0.97	0.91	0.99	0.99	0.89	0.99	0.98	0.96	1		
**ClogP[j]**	0.35	0.91	0.83	0.90	0.89	0.99	0.89	0.93	0.87	0.89	1	
**ClogP[k]**	0.52	0.96	0.91	0.99	0.99	0.88	0.99	0.96	0.97	0.99	0.89	1

[a] clogPS, [b] Molinspirations, [c] OSIRIS property explorer, [d] HyperChem 7.0, [e] Sybyl X, [f] Marvin Sketch (ChemAxon) 15, [g] ChemSketch 2015, [h] Dragon 6.0, [i] Dragon 6.0, [j] Kowwin, [k] XlogP3.

Figure S1. Matrix of correlation coefficients of linear relationships between experimental lipophilicity (log*k*[1]) and calculated lipophilicity with clogPS[2], Molinspirations[3], OSIRIS property explorer[4], HyperChem 7.0[5], Sybyl X[6], Marvin Sketch (ChemAxon) 15[7], ChemSketch 2015[8], Dragon 6.0[9], Dragon 6.0[10], Kowwin[11], XlogP3[12] methods for the entire ensemble of compounds (series A, B, C).

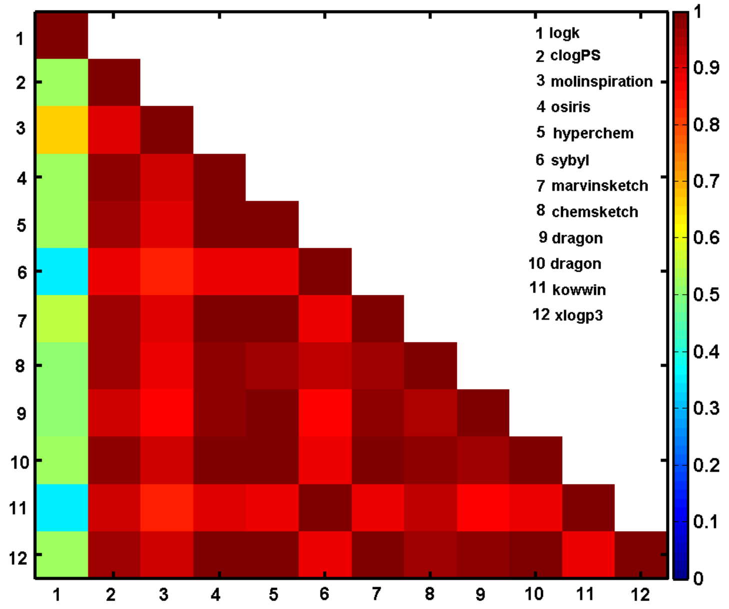

Scheme S1. Basic methods for in silico lipophilicity specification.

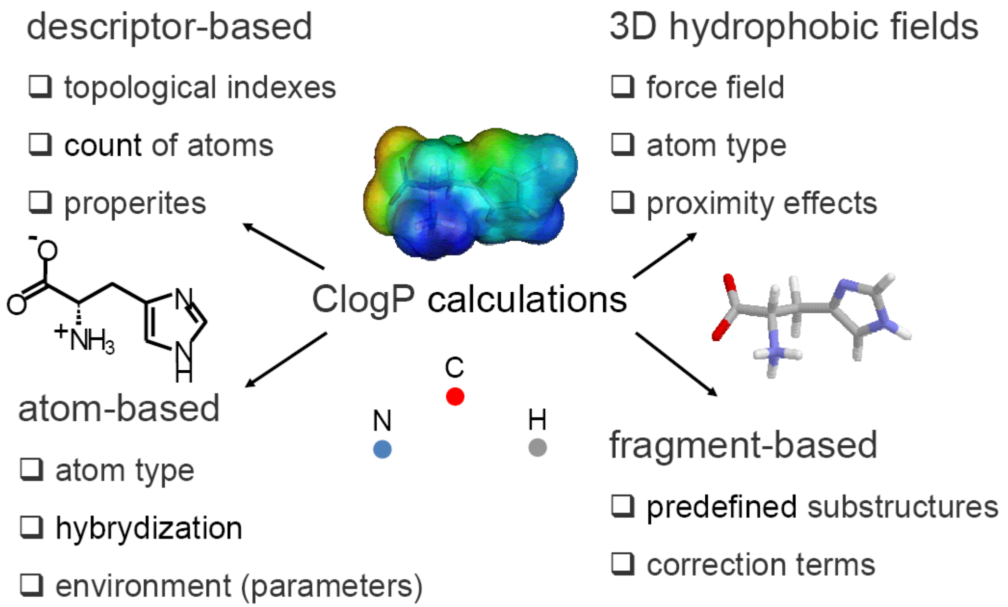

Supplement: Supplementary file 1 [file molecules-23-01635-s001.pdf]
